# Testing the Posterior Chain: Diagnostic Accuracy of the Bunkie Test versus the Isokinetic Hamstrings/Quadriceps Measurement in Patients with Self-Reported Knee Pain and Healthy Controls

**DOI:** 10.3390/jcm13041011

**Published:** 2024-02-09

**Authors:** Anna Gabriel, Andreas Konrad, Nadine Herold, Thomas Horstmann, Robert Schleip, Florian K. Paternoster

**Affiliations:** 1 Department of Conservative and Rehabilitative Orthopedics, School of Medicine and Health, Technical University of Munich, 80992 Munich, Germany; anna-gabriel@tum.de (A.G.); robert.schleip@tum.de (R.S.); 2 Institute of Human Movement Science, Sport and Health, Graz University, 8010 Graz, Austria; 3 Department Sport and Health Sciences, School of Medicine and Health, Technical University of Munich, 80992 Munich, Germany; nadine.herold@tum.de; 4 Department of Medical Professions, Diploma University of Applied Sciences, 37242 Bad Sooden-Allendorf, Germany; 5 Department of Biomechanics in Sports, School of Medicine and Health, Technical University of Munich, 80992 Munich, Germany; florian.paternoster@tum.de

**Keywords:** myofascial diagnostics, myofascial assessments, H/Q ratio, dorsal chain, superficial backline, myofascial chain

## Abstract

(1) **Background**: The isokinetic measurement (IM) of the leg muscles is well established but costly, whereas the Bunkie Test (BT) is a rarely investigated but easy-to-conduct functional test to evaluate the total posterior chain. Although the tests differ in aim and test structures, both have their justification in the assessment process. Therefore, this study evaluated the diagnostic accuracy of the BT and the IM. (2) **Methods**: 21 participants (9 female, 12 male; age, 26.2 ± 5.26 years; weight 73.8 ± 14.6 kg; height 176.0 ± 9.91 cm) and 21 patients (9 female, 12 male; age, 26.5 ± 5.56 years; weight, 72.6 ± 16.9 kg; height 177.0 ± 10.1 cm) with self-reported pain in the knee performed the IM and the BT. For IM, we calculated the ratio of the knee mean flexor/extensor peak torque (H/Q ratio) for 60°/s and 120°/s, and BT performance was measured in seconds. We classified the IM (<0.6 H/Q ratio) and the BT (leg difference ≥4 s) as binary results according to the literature. We calculated the sensitivity and specificity, which we compared with the Chi-Square test, and the 95% confidence intervals (CI). A *p*-value of ≤0.05 is considered significant. (3) **Results**: The sensitivity for the BT was 0.89, 95% CI [0.67, 0.99], and the specificity was 0.52 [0.30, 0.74]. For the IM, the sensitivity was 0.14 [0.03, 0.36] for 60°/s and 0.05 [0.00, 0.24] for 120°/s, and the specificity was 0.70 [0.46, 0.88] for 60°/s and 0.90 [0.68, 0.99] for 120°/s. The results of the Chi-Square tests were significant for the BT (χ^2^ (1) = 6.17, *p* = 0.01) but not for the IM (60°/s: χ^2^ (1) = 0.70, *p* = 0.40; 120°/s: χ^2^ (1) = 0.00, *p* = 0.97). (4) **Conclusions**: Patients were more likely to obtain a positive test result for the BT but not for the IM.

## 1. Introduction

Functional Performance Tests (FPTs) analyze performance aspects, functional abilities, and dysfunctional movement patterns [1,2]. They enable the investigation of diverse physiological functions, including flexibility, endurance, strength, balance, coordination, and motor control, in different body regions [3,4]. Evidence shows that the functionality and integrity of the lower extremity posterior chain (PC) are essential for sports performance [5,6]. The PC comprises the structures of the so-called superficial backline of the lower extremity, namely the myofascial structures of the planar foot, the calf, the dorsal thigh, and the gluteal area. The Bunkie Test (BT) and the isokinetic measurement of the knee muscles are applied to test PC structures [7,8], mainly the hamstrings.

The BT was initially designed to detect imbalances of musculoskeletal chains linked via connective tissue [7] and aims to assess the structures of the PC, mainly the biceps femoris muscle of the tested leg, the gluteal muscles of both sides, and the contralateral back muscles [9]. Therefore, in contrast to other FPTs, which mostly assess isolated muscles or muscle groups, the BT accounts for the fact that muscles generally function in chains with their surrounding connective tissues [10,11]. The test is easy to understand and conduct; it is neither time- nor cost-intensive, and no special equipment is needed [9,12]. The BT is, therefore, frequently applied in daily practice [1,13,14,15]. Nevertheless, the initial study description lacked precise clarification regarding the standardized procedure for conducting the test [7]. Hence, existing studies show differences in the testing procedure, test conduction, and evaluation, which led to incomparable performance results, inconsistent test conduction, and missing normative values. Additionally, there is a shortage of studies that further investigate the quality criteria of the test, such as reliability [1,3,9,13,14,15].

In contrast to the BT, isokinetic testing of the lower extremities is an established and effective method, widely considered a valid ‘gold standard’ and well described in prior studies [16]. Opposed to the BT (i.e., PC), for the isokinetic measurement, the evaluation of muscle strength balance between the knee extensors and flexors during concentric movement is a standard procedure known as the hamstrings/quadriceps ratio (H/Q ratio), defining the ratio of the concentric muscle peak force [17,18,19]. Often, the correct value of the H/Q ratio is a goal to reestablish proper muscle balance and stability of the knee joint [19]. Isokinetic dynamometer measurements are safe, but, in contrast to the BT, they are relatively expensive and require space, time, and expertise [20,21,22], making them often unsuitable for applicability in daily clinical or sports practice [23].

Due to the different advantages, both tests have their own justification for application in screening and assessment. Although the two tests aim to test other structures (isolated muscles versus total PC) and modalities (e.g., peak force versus endurance), both are applied to, i.e., detect potential musculoskeletal dysfunctions in patients with self-reported knee pain. Yet, it is unclear whether the tests show similar results.

Therefore, the main goal of the study is to evaluate the diagnostic accuracy of the two tests in patients with self-reported pain in the lower extremity (i.e., knee area) and healthy controls by comparing the sensitivity and specificity of the index test (i.e., BT) with the standard assessment (i.e., isokinetic measurement). Further, to contribute to the existing body of literature, we additionally report reliability measures of the BT of a preceding test trial. We defined the primary hypothesis as H0: The probability of obtaining a positive test result (defining criteria, see methods) is the same for patients with dysfunctions in the lower extremities and healthy participants for both tests. The secondary hypothesis is H0: The investigated index test (i.e., BT) shows similar results in terms of sensitivity and specificity compared with the standard test (i.e., isokinetic measurement, H/Q ratio).

## 2. Materials and Methods

This cross-sectional study on diagnostic accuracy in an unpaired, between-subjects design [24,25] was conducted in accordance with the Declaration of Helsinki and followed all governmental and hygienic guidelines concerning the COVID-19 pandemic. The university’s ethical committee approved the study protocol (209/21 S-KH). The study was registered in the German Clinical Trials Register (DRKS S00024076). All participants provided written, informed consent before testing. To improve the quality of reporting, the study follows the Statement for Reporting Studies of Diagnostic Accuracy (STARD) [26]. Further, we refer to the studies by Hess et al. [27] and Sitch, Dekkers, Scholefield, and Takwoingi [25] for analyzing and reporting the results. As the study did not receive any additional funding, no funders played a role in the design, conduct, or reporting of this study.

### 2.1. Participants

Patients with self-reported pain in the lower extremities (knee area) (details see Table A1) and healthy participants (all male/female/diverse, 18–40 years old) were included in this study. Patients and healthy participants were all recreationally active (Table 1) [28,29]. Participants and patients were excluded if they had current musculoskeletal pain in the shoulder girdle, the neck, or the elbows and other nonspecific musculoskeletal disorders, e.g., rheumatic disorders. In addition, participants were excluded if they were pregnant, in the nursing period, diagnosed with a neurological disorder, or took medication that affects perception or proprioception. Participants’ characteristics are shown in Table 1.

For estimating the sample size, we assumed—based on our primary hypothesis—that with α = 0.05 and β = 0.8, 80% of the patients with pain can be detected correctly with the index test (sensitivity), and 30% of the healthy participants are considered as such (specificity). This would result in a sample size of 19 healthy participants and 19 patients. With an add-up of 10% to account for potential dropouts, our total sample consisted of 42 participants.

### 2.2. Study Procedure

Patients were allowed to participate in the measurements, consisting of one 60-min session, if they met the inclusion criteria and gave informed consent. The data from the healthy participants was collected in another study (DRKS00027923), where both evaluated tests were performed—amongst others—and included in this study only if participants explicitly agreed on that in the informed consent. Study participants were instructed to avoid heavy physical exercise and alcohol drinking 24 h before the examination. Further, they should not drink caffeine or smoke and refrain from eating heavy meals two hours before the intervention [30]. Participants and patients warmed up with five minutes of cycling at 80 W at a self-selected cadence. Participants performed the two tests in a random order (coin tossing) with a ten-minute break in between [31]. For the participants, the respective first leg assessed was randomly allocated as the reported dominant or non-dominant leg; for the patients, it was always the dominant leg first. The dominant leg was determined as the ‘preferred leg to kick a ball with’. All measurements were conducted and supervised by a trained physiotherapist with more than seven years of practical experience who had already performed the test numerous times, for example, during previous studies on the topic [9,13]. The examiner was blinded concerning the affected leg/side. All participants were blinded concerning the study’s outcome and did not receive further information concerning the respective testing methods a priori.

### 2.3. Measurement

#### 2.3.1. Bunkie Test

**Figure 1:**
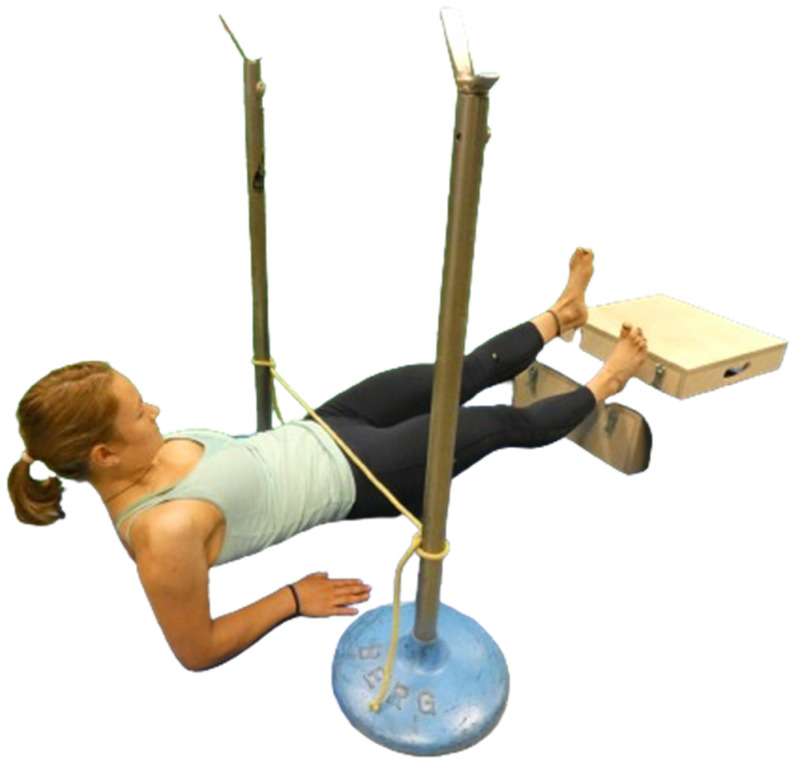
Participants performed a reversed plank and lifted one leg for the standardized version of the Bunkie Test. The optimum height of the pelvis and heel is marked.

We instructed and conducted the standardized version of the BT for the so-called posterior power line, which comprises the structures of the PC, based on our prior study [9] (Figure 1). For the BT, participants placed their forearms on a mat with the shoulders directly above the elbows and their heels on a box with a height of 20 cm, and both legs straightened. Participants were instructed to continue constant breathing during the test, to avoid breath holding, and to immediately report any feeling of fatigue, burning, cramping, pain, or strain in the muscle. To assess the dominant leg, participants lifted their pelvis to a neutral position, marked with a rubber band, stretched horizontally between two fixed stators (Figure 1). Then, they raised the non-dominant foot off the box, where the height was visually referenced with a 10-cm box. Performance was measured in seconds with a stopwatch when the contralateral leg was lifted. The test stopped when the participant either reported any sensation of pain or cramping or ended the test due to fatigue. If participants started to deviate from the neutral standardized body position, they were verbally corrected by the examiner and were allowed to adjust the position once for each body part. The test was halted if there were additional deviations from the neutral position or if participants were unable to either adjust to or sustain the neutral position any longer. After a one-minute pause, the testing procedure was repeated for the contralateral leg [1,3,7,9,15].

#### 2.3.2. Isokinetic Testing

The isokinetic strength testing (Isomed2000, D & R Ferstl GmbH, Hemau, Germany) for the knee flexor and extensor muscles was performed over a range of motion of 90° (0° to 90° knee flexion; 0° = entirely straight leg) at an angular speed of 60°/s and 120°/s. Participants were positioned on the seat (backrest inclination of 10°) and fixed with straps over the shoulders, across the waist, and over the middle of the thigh to avoid unwanted movement. The axis of rotation of the dynamometer was carefully visually aligned with the knee’s axis of rotation. Before the test session, participants got detailed instructions concerning the individual test and did a familiarization trial for each condition, consisting of five submaximal dynamic contractions. For the respective test, for each velocity, one set of five maximal voluntary concentric flexion and extension contractions was performed consecutively. Between each warm-up and test trial, there was a three-minute break. The angle and torque values (Nm) were captured with proEMG at 1000 Hz (prophysics AG, Kloten, Switzerland) and further processed in Matlab (R2020b, The MathWorks Inc., Natick, MA, USA). The mean peak torque value between the second and fourth repetitions (flexion/extension) was used for further analysis. These values were used to calculate the concentric hamstrings/quadriceps ratio (H/Q ratio) [17,32,33,34].

#### 2.3.3. Statistical Analyses

For statistical analysis, the software R (R version 3.5.1) was used [35]. All participants characteristic variables and test data were normally distributed (Shapiro–Wilk test and Levene test for equality of variances). We calculated the mean and standard deviation (SD) and compared the differences between the groups’ participants’ characteristics with the Student’s *t*-test. Further, the effect sizes (Cohen’s d) were calculated and interpreted as d (0.01) = very small, d (0.20) = small, d (0.50) = moderate, d (0.80) = large, d (1.20) = very large, and d (2.00) = huge [36].

For diagnostic accuracy, the statistical models proposed by Knottnerus and Muris [37] were used. Therefore, the main dependent variables are considered binary outcomes. For the BT, one of the primary outcomes is the myofascial performance of the PC, which is measured via performance duration in seconds. For comparability of the tests, we performed a binary classification of the BT, where every outcome with a correct identification of the dysfunctional leg (lower performance value) indicates a positive test result. De Witt and Venter [7] propose that side differences of ≥4 seconds would indicate malfunctioning of the tested myofascial line and therefore have to be considered as clinically relevant outcomes, which we analyzed separately for patients. For participants, such performance differences between legs (≥4 s) were considered a positive test result [7]. Similarly, for the isokinetic measurement, the binary outcome, a H/Q ratio of ≥0.6, was proposed to indicate proper musculoskeletal functioning, as expected in healthy participants, and therefore indicates a negative test result [17,33,34]. If the H/Q ratio of one leg (healthy versus injured for patients and left versus right leg for participants) was ≥0.6 and the other leg < 0.6, this was a positive test result. We further describe and discuss the rationale for the binary classification in the discussion section.

The binary classification results of the BT and the isokinetic measurement test are shown via a 2 × 2 table. The sensitivity, specificity, positive predictive value (PPV), and negative predictive value (NPV), as well as their respective 95% confidence intervals (CI), are calculated for both of the two tests with the epiR package of the statistical software R (R version 3.5.1) [35]. The sensitivity is therefore calculated as A/(A+C), the specificity as D/(B+D), the PPV as A/(A+B), and the NPV as D/(C+D), where A indicates a correct positive, B a false positive, C a false negative, and D a correct negative test result. The sensitivity and specificity are compared with the Chi-Square test with Yates’ continuity correction. A *p*-value of ≤0.05 is considered statistically significant.

## 3. Results

### 3.1. Participant Data

The baseline participants’ characteristics are presented as mean and SD or total numbers (*n*) (Table 1) and did not differ between groups (age: t(21) = −0.150, *p* = 0.88, d = −0.05; weight: t(21) = 0.350, *p* = 0.80, d = 0.08; height: t(21) = −0.309, *p* = 0.76, d = −0.10).

### 3.2. Bunkie Test

The 2 × 2 table of the BT result is shown in Table 2. The calculated sensitivity for the BT is 0.81, 95% CI [0.58, 0.95] (clinically relevant: 0.89 [0.67, 0.99]), the specificity is 0.52 [0.30, 0.74], the PPV is 0.63 [0.42, 0.81], and the NPV is 0.73 [0.45, 0.92] (clinically relevant: 0.85 [0.55, 0.98]). The results of the Chi-Square tests were significant (total data: χ^2^ (1) = 3.73, *p* = 0.05; clinically relevant data: χ^2^ (1) = 6.17, *p* = 0.01).

The patients’ BT results differed significantly between the healthy (21.48 ± 8.74) and injured legs (15.14 ± 7.84) (t(21) = 2.470, *p* = 0.02) with a moderate to large effect size of d = 0.8. For the healthy participants, neither the comparison between the dominant (17 ± 11.0 s) and non-dominant leg (18.6 ± 10.0 s) (t(21) = −0.235, *p* = 0.82, d = −0.1) nor between the first (19.7 ± 10.0) and second tested leg (17.8 ± 10.3) (t(21) = 0.619, *p* = 0.54, d = 0.2) showed significant differences.

### 3.3. Isokinetic Measurement

We excluded one person’s data from the group of healthy participants (P09), as the results showed inconsistency regarding maximum flexion and extension values.

The 2 × 2 table of the isokinetic measurement H/Q ratio results is shown in Table 3. The calculated sensitivity for the isokinetic measurement is 0.14, 95% CI [0.03, 0.36] for 60°/s and 0.05 [0.00, 0.24] for 120°/s; the specificity is 0.70 [0.46, 0.88] for 60°/s and 0.90 [0.68, 0.99] for 120°/s; the PPV is 0.33 [0.07, 0.70] for 60°/s and 0.33 [0.01, 0.91] for 120°/s; the NPV is 0.44 [0.26, 0.62] for 60°/s and 0.47 [0.31, 0.64] for 120°/s. The results of the Chi-Square tests were not significant (60°/s: χ^2^ (1) = 0.70, *p* = 0.40; 120°/s: χ^2^ (1) = 0.00, *p* = 0.97), which means that the probability of obtaining a positive test result is the same for patients with dysfunctions in the lower extremity and healthy participants.

**Figure 2:**
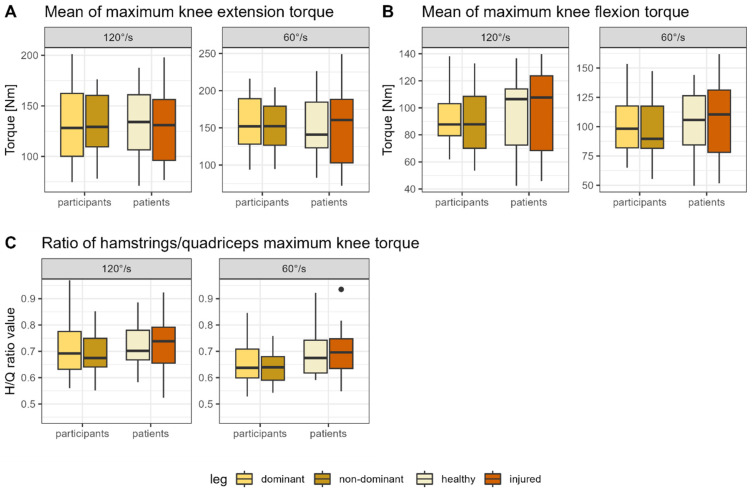
Boxplots showing the mean of the peak concentric (**A**) extension and (**B**) flexion values as well as the (**C**) hamstrings/quadriceps ratio for the isokinetic measurement at 60°/s and 120°/s for the healthy and injured leg (patients) or the dominant and non-dominant leg, respectively (healthy). Note: H/Q ratio, Hamstrings/Quadriceps ratio.

The descriptive test data are shown in Figure 2A–C and additionally listed in Appendix A (Table A2). The analysis revealed that the patients’ results did not differ significantly between the healthy and injured legs for all variables (all *p* > 0.05, d ≤ 0.01) (Table A3). For the healthy participants, the comparison between the dominant and non-dominant legs showed no significantly different results for all parameters (all *p* > 0.05, d = 0.05–0.27) (Table A3). Similarly, there were no significant differences between the first and second tested legs for all parameters (all *p* > 0.05, d = 0.05–0.31) (Table A2).

## 4. Discussion

For the BT, the patients’ results differed significantly between the healthy and injured legs, whereas no differences were detected for the healthy participants. In contrast, neither the participants’ nor the patients’ isokinetic measurement results differed between legs. The results of the Chi-Square tests were only significant for the BT, which means that the probability of obtaining a positive test result is not the same for patients with dysfunctions in the lower extremities and healthy participants. Further, unexpectedly, the results for sensitivity and specificity were better for the index test (BT) compared with the gold standard test (isokinetic measurement).

### 4.1. Diagnostic Accuracy of the Investigated Tests

As proposed by Villafane, Gobbo, Peranzoni, Naik, Imperio, Cleland, and Negrini [12], the validity of our study was defined as the tests’ ability to discriminate between patients with knee pain and healthy participants. Diagnostic accuracy allows a classification of the current health status (e.g., impaired vs. healthy) and is defined as ‘the amount of agreement between the results from the index test and those from the reference standard’ [24,38]. It is, therefore, highly relevant for the practical applicability of the test as an assessment tool [24].

#### 4.1.1. Isokinetic Measurement

In our study, we did not find a difference in H/Q ratios between patients and participants or between the healthy and injured legs in patients using a cut-off value of 0.6. For the H/Q ratio, values between 0.52 and 0.67 are considered optimal. Mandroukas et al. [39] summarize that existing study results for the H/Q ratio vary from 0.5 to 0.83 [8,40]. Grygorowicz, Kubacki, Pilis, Gieremek, and Rzepka [33] provide 0.6 as the normative value at the H/Q ratio for 60°/s, and in their review, Kellis, Sahinis, and Baltzopoulos [8] stated that the conventional ratio values at 60°/s are around 0.6. They found the cut-off values for the traditional ratio at 60°/s varying from 0.47 to 0.66 [8]. A lower limit value than 0.6 led, according to Dauty et al. [41], to fewer predictive possibilities. Although the chosen cut-off point of 0.6 seems to be arguable according to prior literature [8,18,33,39,41,42,43,44], in general, the optimum value for the H/Q ratio depends on angular velocities, meaning the greater the angular velocity, the higher the H/Q ratio value [33,40]. Further, for more functional ratios at 60°/s, the reported H/Q ratio is around 0.8 [8]. Nevertheless, as descriptive test data did not differ greatly between the group of patients and healthy participants, except for the average maximum knee flexion torque at 120°/s, it is unlikely that changes in the chosen cut-off point would have altered the results.

#### 4.1.2. Bunkie Test

De Witt and Venter [7] report that side differences of ≥4 s are noticeable, meaning an increased injury risk, and should be followed by specific rehabilitation and temporary exclusion from professional sports. Nevertheless, the proposed normative values of prior studies vary. De Witt and Venter [7] suggest a typical holding time of 20–40 s, with only endurance athletes likely reaching the 40-s mark. In contrast, Brumitt [1] reports an average value of 40 s for an atypically healthy population. These inconclusive reports are the reason why a standardized test version was recommended in a prior study [9].

### 4.2. Reliability of the Applied Tests

#### 4.2.1. Isokinetic Measurement

The test-retest reliability of the isokinetic measurement for the lower extremity was reported to be good to excellent in previous studies [22,45]. Additionally, Habets et al. [46] reported good intra-rater reliability. For the H/Q ratio specifically, Mau-Moeller et al. [47] reported a moderate-to-high intra-session reliability for conventional ratios.

#### 4.2.2. Bunkie Test

In contrast to the isokinetic measurement, there is a lack of studies investigating the reliability of the BT [9], which is why we investigated the inter-rater reliability in a pre-project (DRKS S00023801). We assessed whether the examiner’s level of experience influences the BT results. Therefore, a physical therapist with ten years of clinical experience and a sports science student of the bachelor program with no further education (both blinded) rated the performance in the BT of 20 participants (healthy (9) or with current pain in the lower limb or back (11)). The inter-rater reliability (ICC3) was calculated for each tested leg separately and was based on a single-rating, consistency, two-way mixed effects model using the software R (version 3.5.1) [48,49,50]. ICC results were classified as <0.50 poor, 0.50–0.75 moderate, 0.75–0.90 good, and >0.90 excellent [49]. To further assess whether the examiners identified participants with dysfunctions correctly, we compared the performance results between the legs with the Wilcoxon signed rank sum test with continuity correction, as the data were not normally distributed, which we tested for with the Shapiro–Wilk test. We included all 20 participants in the final analysis (9 m/11 f; age: 25.8 ± 3.5 years; height: 175.7 ± 8.0 cm; weight: 71.7 ± 12.1 kg). The test results for the left leg (ICC 0.28) and the right leg (ICC 0.14) showed poor inter-rater reliability. The experienced examiner rated the left and right leg significantly differently in patients (*p* = 0.022) but not in healthy participants (*p* = 0.051). In contrast, the students’ ratings did not differ between the legs for patients (*p* = 0.674) and healthy participants (*p* = 0.560), from which we conclude that the experienced examiner correctly identified the injured/dysfunctional side. Therefore, we ensured that participants and patients were rated by the same experienced physical therapist in this study during all measurements.

Although the experienced examiner correctly identified the leg with dysfunction in a population of healthy participants and patients, this was not the case for the inexperienced examiner. In contrast to the isokinetic measurement, the inter-rater reliability was poor. Nevertheless, if an experienced examiner conducts the test, there were significant differences in performance ratings between the healthy and injured legs in patients in this study. In addition, the patients with dysfunctions in the lower extremities were more likely compared with healthy participants to obtain a positive test result, which supports the application of the BT as an additional assessment tool to identify potential dysfunctions in the lower extremities. Further studies should investigate the inter-rater reliability between two examiners with a similar level of experience and the intra-rater reliability.

### 4.3. Comparison of the Two Applied Tests: Testing the Posterior Chain

In this study, we compared two FPTs, which are said to be able to detect injuries and potentially deteriorating motor functions in the area of the PC. Yet, the tests differ in their aim, set-up efforts, and conduct. Therefore, it is interesting to compare the two tests regarding their application as FPTs for the PC. It must be noted that the BT is an isometric holding test, whereas the isokinetic testing in our study was a concentric muscle movement. Further, the BT investigates the total PC, whereas the isokinetic measurement claims to test the knee flexors and extensors’ maximum torque value precisely.

An advantage of the BT over the isokinetic measurement is that it tests various connected PC muscles, which are activated during the test [9]. Currently, commonly applied isometric trunk holding tests were designed and are validated, similar to the isokinetic measurement of the knee flexors, to test only specific muscles (e.g., the Sorensen test for evaluating the back extensor muscles and predicting low back pain [12,51,52,53], or the prone bridging test for the abdominal muscles [54]). Nevertheless, current findings that muscles work together in chains linked via the surrounding connective tissue [10] and co-activation of the PC muscles must be taken into account [10,11] (i.e., also during the Sorensen test, there is co-activation of the gluteal and the hamstrings muscles [51]). This leads to the assumption that the BT might cover findings that are not considered by the isokinetic measurement. This could explain the differences between the tests found in our study.

Another promising, recently proposed test is the standing 90:20 isometric posterior chain test, which evaluates the applied force of the total chain on a pressure plate [55,56]. Still, both the BT and the standing 90:20 isometric posterior chain test lack standardized assessment concerning the interpretation of the results (i.e., norm values) and comparison studies with well-established performance tests. In addition, we would recommend that further studies investigate other injuries and orthopedic disorders of the PC, e.g., ankle sprain or hamstring strain.

### 4.4. Limitations

We are aware that this study has several limitations. First, there might be some form of spectrum and selection bias, which occurs when there are, e.g., more advanced cases in the population than in the study sample. There might have also been light prior injuries or myofascial dysfunctions in healthy participants, which they might not have noticed and would be against classifying them as ‘healthy’ [37]. Yet, this is closely linked to screening and assessment in daily practice, where there is never a completely clear distinction between healthy persons and persons with, i.e., slowly progressing myofascial imbalances over time.

In our study, we mostly referred to ‘soft’ measures when categorizing patients and healthy participants. Although Knottnerus and Muris [37] recommend additionally including ‘harder’ investigations (e.g., X-rays) in diagnostic studies, it is common in daily clinical and sports practice that therapists and practitioners cannot refer to such additional diagnostic material. Further, for most non-traumatic orthopedic diseases (i.e., low back pain), such ‘hard investigations’ (i.e., imaging techniques) are not recommended in the first line by clinical guidelines [57], as symptoms and objective findings often do not match [58]. By referring to self-reported measures instead (i.e., pain), we additionally addressed observer bias, which depends on physicians’ ability to accurately detect, e.g., a history of an ankle sprain [37]. For inter- and intra-individual test comparison, when assessing the force of the lower extremities’ PC, it is not only recommended to differentiate between the dominant and the non-dominant leg but also to standardize the test results with regard to the person’s body weight [59,60]. As there is a lack of proposed procedures for standardization, at least for the BT, we did not apply this procedure.

We are aware that there is insufficient data concerning the reliability of the BT. We addressed concerns regarding inter-rater reliability by choosing one experienced examiner to evaluate the test. Missing data concerning the intra-rater and inter-measurement reliability of the BT must be considered a limitation when interpreting the results of this study.

In addition, it could have been advantageous for the H/Q ratio to prefer a specific range instead of 0.6 as the cut-off value. Yet, we chose 4 s (no range) as the cut-off value for the BT. Therefore, to increase comparability, we decided to go for a cut-off value (i.e., 0.6) instead of a cut-off range for the isokinetic measurement, too.

### 4.5. Clinical Implications

The BT serves as a FPT that proves to be easily applicable in both routine clinical and sports practices due to its minimal resource requirements and time efficiency. Despite its frequent utilization, the BT lacks comprehensive, high-quality evaluation studies. In contrast, isokinetic measurement is currently considered the gold standard, providing precise data that aligns with a vast body of prior research. However, its applicability in professional sports settings is hindered by its reliance on expensive equipment and specialized expertise.

This study demonstrates that the BT may present a straightforward, cost-effective, and time-efficient alternative for assessing potential dysfunctions in the lower extremity. Consequently, athletic trainers, physiotherapists, sports scientists, or medical doctors may incorporate this test in screening, evaluating rehabilitation progress, and making decisions regarding the return to sports. It is crucial to emphasize that accurate BT assessment demands expertise. Furthermore, none of the examined tests should be used as stand-alone diagnostic criteria; instead, they should be regarded as additional tools in the overall assessment process.

## 5. Conclusions

In this study, the BT showed promising results on sensitivity and specificity, as the test performance results for patients with self-reported knee pain differed significantly between the healthy and the injured leg. The probability of obtaining a positive test result differed for patients with dysfunctions in the lower extremities and healthy participants. Nevertheless, we think that the BT must be conducted in the standardized test version and rated by an experienced examiner. The lack of tests assessing the total PC should be addressed by further research.

## Figures and Tables

**Table 1:** Participants’ characteristics.

	Patients	Healthy Controls
Sex (*n*)	9 f/12 m ^1^	9 f/12 m ^1^
Age (years)	26.5 (5.7)	26.2 (5.3)
Weight (kg)	72.6 16.9)	73.8 (14.6)
Height (cm)	177.0 (10.1)	176.0 (9.9)
Dominant leg (*n*)	19r/2l ^2^	19r/2l ^2^
Activity status (min/week)	250 (30) ^3^	230 (70) ^3^

^1^ f = female, m = male; ^2^ r = right; l = left; ^3^ moderate physical activity in minutes or doubled amount of vigorous activity [28,29]; Note: Data presented as mean (SD) or total numbers (*n*).

**Table 2:** Binary results for the Bunkie Test.

Test Result	Patients	Healthy Controls
Positive	A: 17 (17 *)	B: 10 (10 *)
Negative	C: 4 (2 *)	D: 11 (11 *)

Note: A: Correct positive; B: False positive; C: False negative; D: Correct negative; *, the numbers in brackets indicate the proportion of clinically relevant results (leg difference ≥ 4 s).

**Table 3:** Binary results for the isokinetic measurement of the lower extremity.

Test Result 60°/s	Patient	Healthy Controls
Positive	A: 3	B: 6
Negative	C: 18	D: 14
**Test Result 120°/s**		
Positive	A: 1	B: 2
Negative	C: 20	D: 18

Note: Participants positive result: Hamstrings/Quadriceps ratio one leg positive, other negative (left and right) Patients positive result: Hamstrings/Quadriceps ratio injured leg positive; A: Correct positive; B: False positive; C: False negative; D: Correct negative.

## Data Availability

Research data are available on request.

## Appendix A

**Table A1:** Description of details concerning the self-reported pathology of patients.

Participant	Affected Side	Reported Pathology	Start/time of Complaints (Years)
01	Dominant	Pain after conservative treatment after knee cartilage injury	1
02	Dominant	Knee pain in the patella region after sports activity or during sitting	5
03	Non-dominant	Pain due to inflammation of the bone skin in the knee area	0.5
04	Dominant	Pain after anterior ligament rupture	1
05	Non-dominant	Knee pain due to restricted hip rotation	0.5
06	dominant	Pain medial knee joint	6
07	dominant	Suspected meniscal tear after slipping on ice (as child), but no assured diagnose, pain started again this year	0.5
08	Non-dominant	Pain due to Edema of the bone marrow in the knee	0.5
09	Dominant	Pain medial and lateral of patella, especially during volleyball	0.5
10	Dominant	Pain resulting from overload of patellar tendon	0.5
11	Dominant	Pain after patella luxation	0.5
12	Dominant	Muscle pain in the thigh (maybe strain)	3
13	Non-dominant	Knee pain following functional foot deformity	3
14	Dominant	Muscle strength deficits after anterior cruciate ligament rupture	2
15	Dominant	Range of motion deficit after anterior cruciate ligament rupture	0.5
16	Non-dominant	Problem with medial meniscus due to overload (sports)	4
17	Non-dominant	Functional deficits following anterior cruciate ligament rupture surgery	6
18	Non-dominant	Instability and pain following knee surgery (anterior cruciate ligament and meniscus)	6
19	Non-dominant	Pain in the patella region after accident	2
20	Non-dominant	Knee pain after ankle sprain	6
21	Non-dominant	Lateral knee pain, especially during sports	7

**Table A2:** Descriptive test data for the maximum extension/flexion value, the mean of the five maximum flexion/extension values, and the ratio of the maximum flexion/extension values of the isokinetic measurement for patients and healthy participants.

Group	Tested Leg	Tested Variable	60°/s [N]	120°/s [N]
Patients	Healthy	Maximum extension	160.4 ± 48.0	135.18 ± 37.1
Patients	Healthy	Maximum flexion	107.6 ± 29.6	98.7 ± 29.3
Patients	Healthy	Mean extension	152.0 ± 45.9	131.5 ± 37.8
Patients	Healthy	Mean Flexion	104.5 ± 33.1	95.6 ± 29.0
Patients	Healthy	Ratio maximum flexion/extension	0.70 ± 0.11	0.73 ± 0.09
Patients	Injured	Maximum extension	235.5 ± 50.4	134.4 ± 39.6
Patients	Injured	Maximum flexion	105.4 ± 35.7	99.8 ± 33.6
Patients	Injured	Mean extension	147.8 ± 49.0	130.2 ± 38.9
Patients	Injured	Mean Flexion	101.8 ± 39.3	95.6 ± 33.0
Patients	Injured	Ratio maximum flexion/extension	0.69 ± 0.10	0.73 ± 0.12
Participants	dominant	Maximum extension	168.8 ± 36.3	141.8 ± 37.7
Participants	Dominant	Maximum flexion	109.2 ± 24.1	99.3 ± 21.7
Participants	Dominant	Mean extension	159.8 ± 35.9	136.9 ± 31.7
Participants	Dominant	Mean Flexion	104.8 ± 24.2	94.9 ± 22.0
Participants	Dominant	Ratio maximum flexion/extension	0.66 ± 0.08	0.70 ± 0.10
Participants	Non-dominant	Maximum extension	162.6 ± 36.9	136.0 ± 35.2
Participants	Non-dominant	Maximum flexion	102.1 ± 24.5	92.3 ± 22.1
Participants	Non-dominant	Mean extension	155.1 ± 34.4	131.8 ± 34.6
Participants	Non-dominant	Mean Flexion	98.3 ± 24.5	89.5 ± 22.5
Participants	Non-dominant	Ratio maximum flexion/extension	0.63 ± 0.06	0.69 ± 0.15

Note: Mean flexion and mean extension indicate the mean of the maximum value of movement repetition two to four of five repetitions; for patients, the dominant leg was always tested first.

**Table A3:** Results of the statistical comparison between tested legs for the isokinetic measurement.

Group	Comparison	°/s	Tested Variable	Result Statistical Analysis
Patients	Healthy/injured leg	60°	Maximum flexion	t(21) = 0.099, *p* = 0.92, d = 0.031
		60°	Maximum extension	t(21) = 0.007, *p* = 1.00, d = 0.002
		60°	H/Q ratio	t(21) = 0.052, *p* = 0.96, d = 0.016
		120°	Maximum flexion	t(21) = 0.036, *p* = 0.97, d = 0.011
		120°	Maximum extension	t(21) = −0.145, *p* = 0.89, d = −0.045
		120°	H/Q ratio	t(21) = −0.279, *p* = 0.77, d = −0.092
	Dominant/non-dominant leg	60°	Maximum flexion	t(21) = 0.724, *p* = 0.47, d = 0.223
		60°	Maximum extension	t(21) = 0.542, *p* = 0.59, d = 0.167
		60°	H/Q ratio	t(21) = 0.395, *p* = 0.70, d = 0.122
		120°	Maximum flexion	t(21) = 0.440, *p* = 0.66, d = 0.136
		120°	Maximum extension	t(21) = 0.221, *p* = 0.83, d = 0.068
		120°	H/Q ratio	t(21) = 0.609, *p* = 0.55, d = 0.188
Participants	Dominant/non-dominant leg	60°	Maximum flexion	t(20) = 0.464, *p* = 0.65, d = 0.147
		60°	Maximum extension	t(20) = 0.293, *p* = 0.77, d = 0.093
		60°	H/Q ratio	t(20) = 0.715, *p* = 0.48, d = 0.226
		120°	Maximum flexion	t(20) = 0.410, *p* = 0.68, d = 0.130
		120°	Maximum extension	t(20) = 0.165, *p* = 0.87, d = 0.052
		120°	H/Q ratio	t(20) = 0.858, *p* = 0.40, d = 0.271
	First-/second tested leg	60°	Maximum flexion	t(20) = 0.845, *p* = 0.40, d = 0.267
		60°	Maximum extension	t(20) = 0.233, *p* = 0.82, d = 0.074
		60°	H/Q ratio	t(20) = 0.994, *p* = 0.33, d = 0.314
		120°	Maximum flexion	t(20) = 0.551, *p* = 0.59, d = 0.174
		120°	Maximum extension	t(20) = 0.379, *p* = 0.71, d = 0.120
		120°	H/Q ratio	t(20) = 0.151, *p* = 0.88, d = 0.048

Note: H/Q ratio, Hamstrings/Quadriceps ratio; Mean flexion and mean extension indicate the mean of the maximum value of movement repetitions of two to four of five repetitions.
